# A Case of Antibody-Mediated Recurrent Hypoglycemia in a Patient With Mixed Connective Tissue Disease

**DOI:** 10.7759/cureus.102277

**Published:** 2026-01-25

**Authors:** Niyati Patel, Hamza Choudhry, Zehra Rahman, Kaitlyn N Romero, Lauren Hassan, Payal Shooliz

**Affiliations:** 1 Internal Medicine, University of Florida College of Medicine, Jacksonville, USA; 2 Endocrinology, University of Florida College of Medicine, Jacksonville, USA

**Keywords:** autoimmune insulin syndrome, hydroxychloroquine, hyperinsulinemic hypoglycemia, insulin autoantibodies, symptomatic hypoglycemia

## Abstract

Insulin Autoimmune Syndrome (IAS) is a rare autoimmune condition in which the body produces antibodies to insulin, leading to recurrent episodes of severe hypoglycemia accompanied by elevated serum insulin and insulin antibody levels. Hypoglycemia can mimic neurological conditions such as stroke. Also known as Hirata’s disease, IAS occurs in patients with autoimmune conditions or a genetic predisposition. IAS is typically treated with immunosuppressive agents, including steroids and rituximab, and patients often undergo thorough evaluations to rule out more common causes of hypoglycemia. Here, we present a case of a patient with pulmonary arterial hypertension (type 1) and mixed connective tissue disease who experienced persistent and recurrent symptomatic hypoglycemia, with glucose levels as low as 35 mg/dL (1.94 mmol/L). C-peptide, a marker of endogenous insulin production, was within normal limits at 2.9 ng/mL (0.96 nmol/L) while the patient was hypoglycemic (glucose <60 mg/dL (3.3 mmol/L)), indicating inappropriate insulin secretion. Total insulin levels were markedly elevated at 7,000 µU/mL (48,615.00 pmol/L), and free insulin levels were elevated at 24 µU/mL (166.68 pmol/L), which initially raised concern for an insulinoma. However, insulin antibodies were evaluated and found to be significantly elevated at >625 µU/mL (>4,340.63 pmol/L), confirming the diagnosis of Hirata’s disease. The patient improved following dietary modifications, initiation of prednisone, and discontinuation of home hydroxychloroquine.

## Introduction

Insulin Autoimmune Syndrome (IAS) leads to immune-mediated hypoglycemia through the production of insulin autoantibodies (IAA). Spontaneous IAS is most common in the Japanese population, as they are more likely to express HLA-DRB1*0406, an allele associated with the development of IAS. Most cases in non-Japanese populations occur in Caucasians, with rare cases documented in African American women, such as in our patient [1]. When IAS is diagnosed in a non-Japanese patient, viruses, medications, or underlying medical conditions are often implicated. Viruses may mimic antigens, leading to the production of IAA and the subsequent development of IAS. Additionally, medications containing sulfhydryl groups have been associated with IAS. About 80% of IAS cases are seen in patients with pre-existing autoimmune conditions, including Graves’ disease, systemic lupus erythematosus (SLE), rheumatoid arthritis, and chronic hepatitis [2].

IAS is caused by a type VII hypersensitivity reaction, in which IAA, typically Immunoglobulin G (IgG), binds to insulin, forming immune complexes. IAA have a high binding capacity for insulin, allowing a single antibody to form complexes with multiple insulin molecules. However, due to the antibodies’ low affinity, these complexes frequently dissociate, increasing the levels of free plasma insulin, even during periods of low plasma glucose. IAS typically presents with symptomatic hypoglycemia during fasting and hyperglycemia postprandially. When plasma glucose levels rise, pancreatic beta cells secrete insulin. IAA bind to this insulin, preventing it from exerting its physiological effects and resulting in decreased levels of free insulin and postprandial hyperglycemia. This hyperglycemia then stimulates further insulin secretion, some of which is not bound by IAA and can function normally. Simultaneously, the low-affinity IAA-insulin complexes begin to dissociate, leading to an excess of free insulin in the bloodstream despite low plasma glucose levels. These concurrent processes result in a surge of free insulin during hypoglycemia. As a result, patients present with the Whipple triad, which includes serum glucose <55 mg/dL, symptoms of hypoglycemia, and symptom resolution following an increase in serum glucose [2].

This article was previously presented as a meeting abstract at the 2025 Southern Regional Meeting on February 13, 2025.

## Case presentation

**Figure 1:**
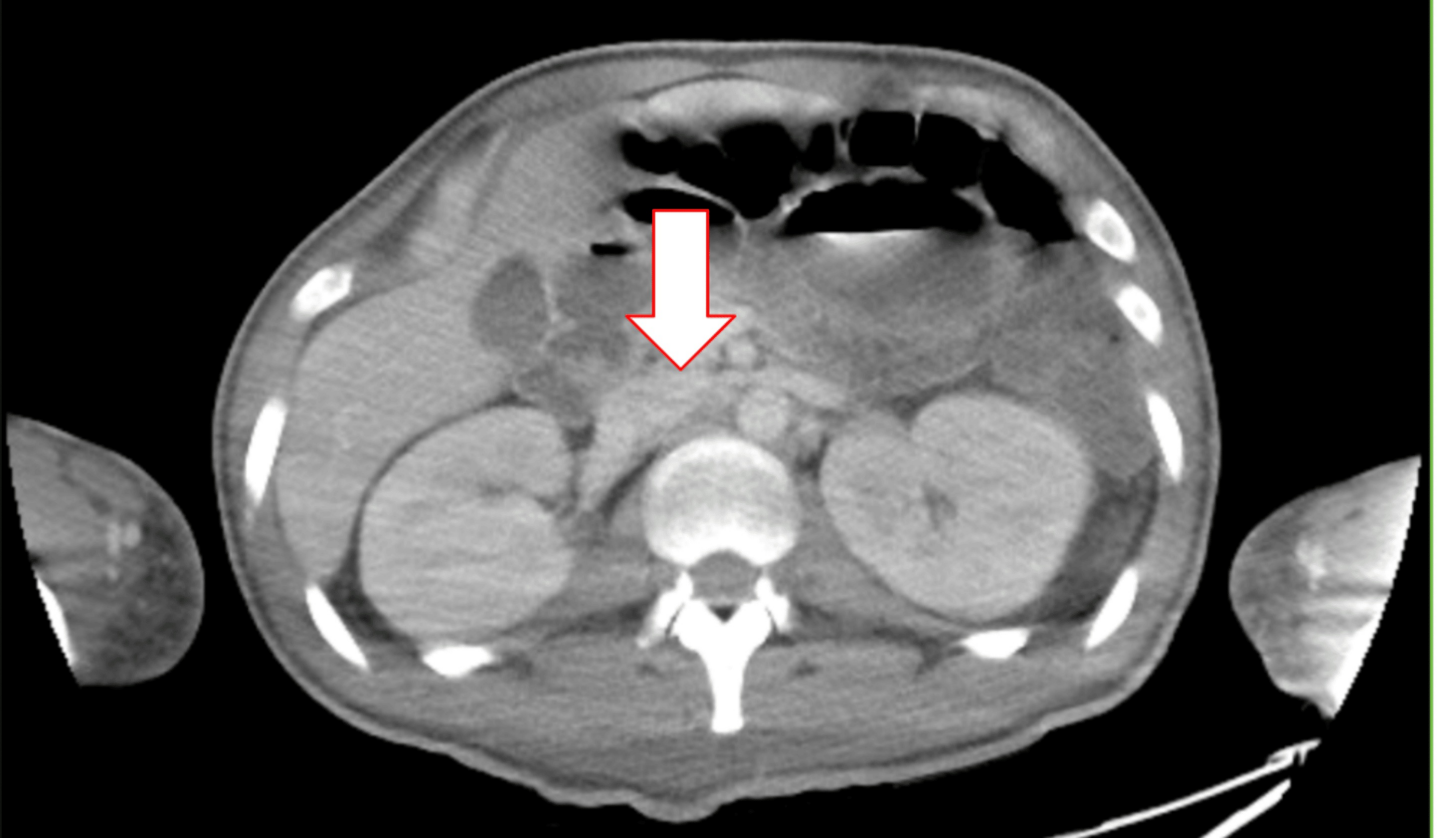
CT abdomen and pelvis (axial view), obtained prior to IAA results returning positive, with the arrow pointing to the pancreas; no insulinoma is present. IAA: Insulin autoantibodies.

**Figure 2:**
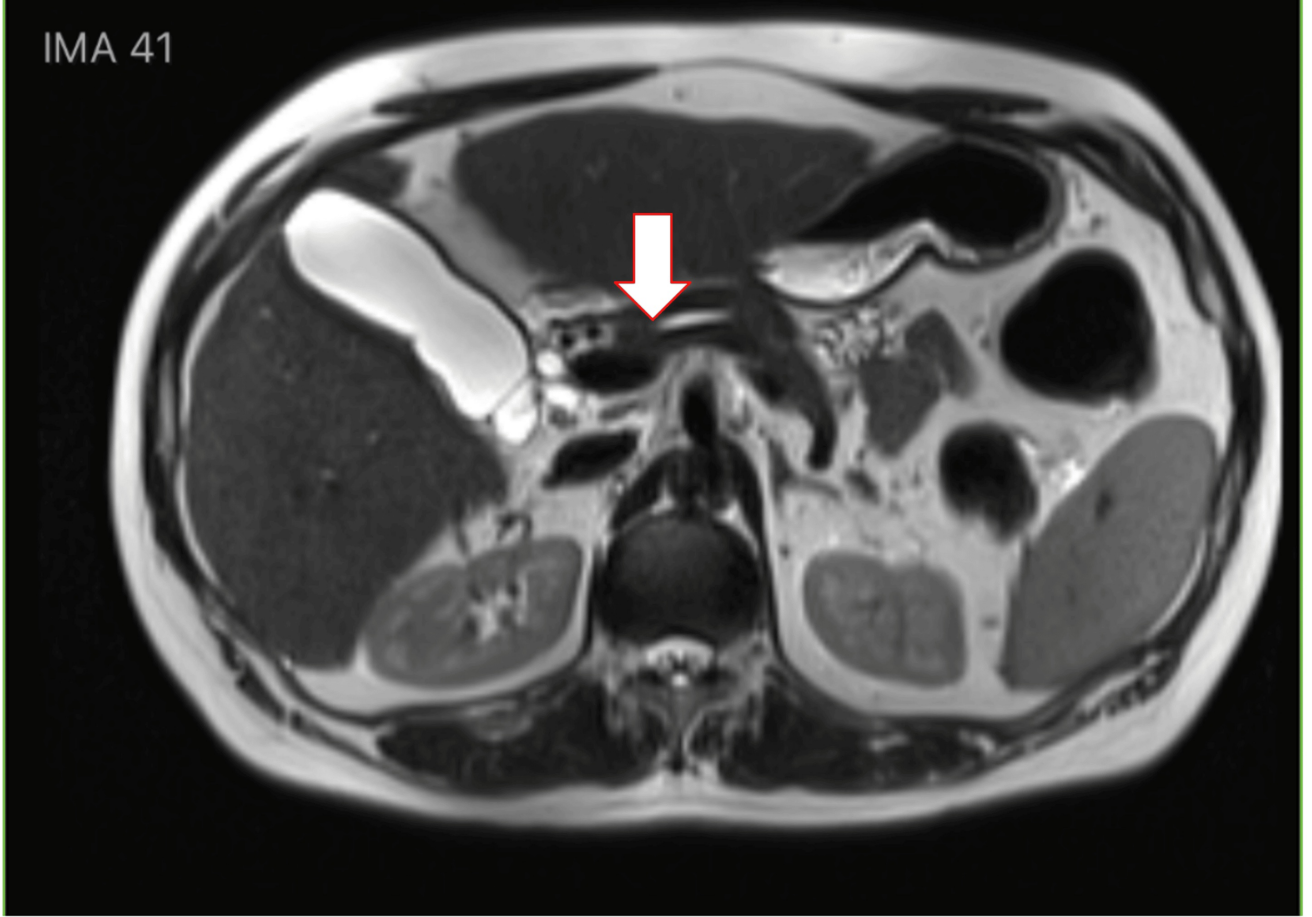
MRCP demonstrating no insulinoma in the pancreas. MRCP: Magnetic resonance cholangiopancreatography.

A 65-year-old female with a medical history of mixed connective tissue disease, including SLE, pulmonary hypertension, hypothyroidism, and heart failure, presented to the hospital after a fall and complaints of shortness of breath. The patient was found to be in acute hypoxic respiratory failure, which resolved with the initiation of sildenafil for pulmonary hypertension. The patient's hospital course was complicated by episodes of postprandial hyperglycemia and symptomatic fasting hypoglycemia, which resolved after administration of glucose tablets or intravenous dextrose. CT of the abdomen and pelvis is displayed in Figure 1, and magnetic resonance cholangiopancreatography (MRCP) is displayed in Figure 2. These images were obtained prior to IAA returning positive and were negative for an insulinoma, helping to rule out an insulinoma. Endocrinology was consulted for the recurrent episodes of symptomatic hypoglycemia and recommended a 72-hour fasting test with accu-checks every two hours. Within 12 hours of fasting, the patient’s blood glucose level dropped to 42 mg/dL, as measured by accu-check. Laboratory tests, displayed in Table 1, were immediately collected at that time, followed by administration of glucose tablets.

**Table 1:** All hypoglycemia laboratory workup was collected 12 hours into the patient’s 72-hour fast, when plasma glucose was 50 mg/dL (2.77 mmol/L). C-peptide: Connecting peptide; IGF: Insulin-like growth factor; GFR: Glomerular filtration rate.

Lab	Result	Reference Range
Plasma glucose after 12-hour fast	50 mg/dL (2.77 mmol/L)	71-99 mg/dL (3.94-5.49 mmol/L)
Free insulin	24 µIU/mL (166.68 pmol/L)	0-17 µIU/mL (0-118.06 pmol/L)
Total insulin	7,000 µIU/mL (48,615.00 pmol/L)	Represents free and bound insulin
C-peptide	2.4 ng/mL (0.96 nmol/L)	1.1-4.4 ng/mL (fasting) (0.36-1.46 nmol/L)
Proinsulin	0.9 µIU/mL (6.25 pmol/L)	0-1.44 µIU/mL (0.0-10.0 pmol/L)
Insulin antibodies	>625 µIU/mL (>4,340.63 pmol/L)	<5.0 µIU/mL is negative (<34.73 pmol/L)
IGF-1	73 ng/mL (9.56 nmol/L)	57-202 ng/mL (7.47-26.46 nmol/L)
Beta-hydroxybutyrate	0.7 mg/dL (67.27 µmol/L)	0.2-2.8 mg/dL (19.21-268.97 µmol/L)
Morning cortisol	9.1 µg/dL (251.05 nmol/L)	6-23 µg/dL (165.53-634.52 nmol/L)
Sulfonylurea panel	Negative	Negative
Aspartate aminotransferase	32 U/L (0.53 µkat/L)	8-48 U/L (0.13-0.80 µkat/L)
Alanine aminotransferase	15 U/L (0.25 µkat/L)	7-55 U/L (0.12-0.92 µkat/L)
Alkaline phosphatase	45 U/L (0.75 µkat/L)	40-129 U/L (0.67-2.15 µkat/L)
Albumin	3.0 g/dL (30 g/L)	3.5-5.2 g/dL (35-52 g/L)
Total protein	6.7 g/dL (67 g/L)	6.3-7.9 g/dL (63-79 g/L)
Potassium	3.7 mEq/L (3.7 mmol/L)	3.5-5.1 mEq/L (3.5-5.1 mmol/L)
Sodium	141 mEq/L (141 mmol/L)	136-145 mEq/L (136-145 mmol/L)
Chloride	101 mEq/L (101 mmol/L)	98-107 mEq/L (98-107 mmol/L)
Anion gap	5 mEq/L (5 mmol/L)	4-16 mEq/L (4-16 mmol/L)
Creatinine	0.95 mg/dL (83.98 µmol/L)	0.6-1.1 mg/dL (53-97.2 µmol/L)
Blood urea nitrogen	14 mg/dL (5 mmol/L)	10-20 mg/dL (3.6-7.1 mmol/L)
Bicarbonate	34 mEq/L (34 mmol/L)	23-29 mEq/L (23-29 mmol/L)
GFR	66 mL/min/1.73 m²	≥60 mL/min/1.73 m²

Laboratory workup revealed that the patient had hyperinsulinemic-mediated hypoglycemia. In patients with this condition, a C-peptide level less than 0.2 ng/mL (0.07 nmol/L) and a proinsulin level less than 0.72 µU/mL (5 pmol/L) (reference range: 0-1.44 µU/mL; 0.0-10.0 pmol/L) suggest exogenous insulin-mediated hypoglycemia. In contrast, patients with endogenous insulin-mediated hypoglycemia will typically have C-peptide levels greater than 0.2 ng/mL and proinsulin levels greater than 0.72 µU/mL. In this subgroup, it is essential to rule out hypoglycemia caused by sulfonylureas or meglitinides by testing serum levels for these agents. Once those causes are excluded, the differential narrows to IAS or insulinoma. Both conditions present with fasting hypoglycemia; however, only IAS presents with concomitant postprandial hyperglycemia.

Laboratory findings in insulinoma typically reveal insulin levels less than 1,000 µU/mL (48,615.00 pmol/L), significantly elevated C-peptide levels, and negative insulin antibodies. Abdominal CT imaging usually reveals an insulinoma, while imaging is not indicated in IAS. Differentiating between IAS and insulinoma is crucial, as the definitive treatment for insulinoma is surgical resection [3].

Notably, insulin-like growth factor 1 (IGF-1) levels were assessed to evaluate for non-islet cell tumor hypoglycemia (NICTH), a paraneoplastic syndrome in which tumors overproduce IGF-2. Excess IGF-2 mimics insulin activity, causing fasting hypoglycemia and suppressing growth hormone secretion, which consequently results in reduced IGF-1 levels.

The patient’s use of hydroxychloroquine was suspected to contribute to her hypoglycemia [4]. Although hydroxychloroquine is not known to directly trigger IAS, it increases the half-life of insulin and inhibits the enzyme responsible for insulin degradation. In patients with IAS, who already have inappropriately elevated insulin levels during fasting, this medication may increase the frequency and severity of hypoglycemic episodes. Moreover, even in patients without IAS, hydroxychloroquine can contribute to fasting hypoglycemia by inhibiting glutamate dehydrogenase activity, a key enzyme in gluconeogenesis [5]. After discontinuing hydroxychloroquine, the frequency and severity of our patient’s fasting hypoglycemia significantly improved.

In our patient, diazoxide was initially not considered the best therapeutic option due to her concomitant diagnosis of pulmonary hypertension and the associated risk of fluid retention. She was first trialed on subcutaneous octreotide 25 micrograms (mcg) once daily, which was up-titrated to 50 mcg twice daily. However, despite these adjustments, she continued to experience episodes of fasting hypoglycemia. She was then trialed on diazoxide 75 milligrams (mg) once daily, but did not respond appropriately. The risk of fluid retention was mitigated by following dosing guidelines from prior studies, which indicate that 2-3 mg/kg/day is both effective and generally well tolerated for the treatment of hyperinsulinemic hypoglycemia. For this patient, weighing 91 kg, the prescribed dose fell within the recommended range. However, due to persistent hypoglycemic episodes, the medication was ultimately discontinued [6].

Following discontinuation of hydroxychloroquine, an improvement in the frequency and severity of fasting hypoglycemia was observed; however, this occurred in the context of concurrent interventions including steroid tapering and dietary adjustments. Ultimately, the patient responded well to prednisone 20 mg and dietary modifications involving low-carbohydrate meals. However, she was later readmitted for seizures caused by symptomatic hypoglycemia, which occurred after missing multiple doses of prednisone. At that time, insulin antibody levels remained unchanged. She was discharged on prednisone 20 mg with a plan to taper the dose every two weeks until reaching the lowest effective dose to prevent hypoglycemia. A continuous glucose monitor (CGM) was also placed. Upon review of the CGM data, the patient experienced fasting hypoglycemic events after tapering to 10 mg of prednisone, as well as episodes of postprandial hyperglycemia, with an average glucose of 166 mg/dL (8.05 mmol/L) and an elevated hemoglobin A1C of 10.6%. As a result, prednisone was increased to 15 mg, with plans to taper every three weeks to 10 mg, followed by 7.5 mg, as long as no further hypoglycemic episodes occurred. She was also initiated on metformin 500 mg twice daily. Metformin was initiated in this patient based on evidence from the American Diabetes Association and the European Association for the Study of Diabetes indicating that metformin monotherapy carries a low risk of hypoglycemia. This favorable safety profile is attributable to its primary mechanisms of action, which include suppression of hepatic gluconeogenesis and increased peripheral glucose uptake through greater insulin sensitivity, rendering hypoglycemia uncommon when used alone [7].

## Discussion

Patients with IAS typically have insulin levels greater than 1,000 µU/mL (48,615.00 pmol/L), with disproportionately lower or normal C-peptide levels and positive IAA, as seen in our patient. Total insulin is typically greater than free insulin due to the presence of IAA. Even prior to a positive IAA result, clinical suspicion was high due to her risk factors, being female and having a pre-existing autoimmune disorder. Although a positive IAA test is highly specific for IAS, current assays detect only IgG-mediated antibodies. In our patient, additional testing was not pursued given the positive IAA result. However, in patients with negative IAA findings but a high clinical suspicion for IAS, and after exclusion of other causes of hypoglycemia, further evaluation is warranted using polyethylene glycol (PEG) precipitation and gel filtration chromatography. In PEG precipitation, insulin-antibody complexes precipitate out of solution, resulting in a reduction in measurable total insulin. This decrease indicates the presence of insulin-binding antibodies. However, this assay is temperature-sensitive, and the presence of other large non-insulin molecules may affect its accuracy. Gel filtration chromatography, on the other hand, separates insulin-antibody complexes from free insulin based on molecular size and serves as a confirmatory test, although it is more costly and time-consuming [8].

In Japanese patients, IAS frequently self-resolves within months after discontinuation of the trigger medication. Even in non-Asian patients, removal of the offending drug and lifestyle modifications have been effective in resolving IAS, as evidenced by decreasing or undetectable IAA levels. Lifestyle modifications include eating small, frequent, low-carbohydrate meals to prevent postprandial hyperglycemia, which can lead to insulin spikes and subsequent hypoglycemic episodes. Meals containing cornstarch have also been shown to be effective, as cornstarch is slowly absorbed in the small intestine, reducing postprandial insulin spikes. Similarly, acarbose, an alpha-glucosidase inhibitor, slows carbohydrate absorption and blunts postprandial insulin elevations [9]. Patients who do not respond to lifestyle modifications and drug withdrawal often benefit from high-dose corticosteroids due to the autoimmune nature of IAS. In more refractory cases, immunosuppressive agents such as rituximab or azathioprine are required. Rituximab targets CD20 receptors on B lymphocytes, reducing the production of IAA, while azathioprine prevents the formation of new autoantibodies, helping maintain remission [10]. In severe or refractory cases, plasmapheresis may be used to rapidly decrease circulating antibody levels. Other treatments that help reduce total insulin levels include somatostatin analogues, diazoxide, and, in very rare cases, pancreatectomy [11].

For all patients, a CGM is an essential tool in managing IAS. CGMs track glucose levels every five minutes, allowing patients to receive alerts before the onset of symptoms, thereby helping to prevent life-threatening complications [12].

## Conclusions

This case underscores the importance of considering IAS in the differential diagnosis of recurrent hypoglycemia, particularly in patients with underlying autoimmune disease, and recognizing hydroxychloroquine as a rare potential contributor to hypoglycemia. IAS is characterized by fasting hypoglycemia with postprandial hyperglycemia and can be distinguished from insulinoma by markedly elevated total insulin levels (>1,000 µU/mL), inappropriately low or normal C-peptide levels, and positive IAA. In this patient, discontinuation of hydroxychloroquine, along with the addition of steroids and dietary modifications, led to a significant reduction in the frequency and severity of hypoglycemic episodes, suggesting a contributory role in hypoglycemia despite not being a direct cause of IAS. Management of IAS centers on identifying and removing potential triggers and implementing lifestyle interventions such as small, frequent, low-carbohydrate meals. Given the rarity of IAS and the lack of standardized treatment guidelines, management should be individualized, and further research is needed to inform evidence-based diagnostic and therapeutic strategies.
